# Underwater sound production of free-ranging Hawaiian monk seals

**DOI:** 10.1098/rsos.250987

**Published:** 2025-11-12

**Authors:** Kirby Parnell, Caroline Smith, Adriana Diaz, Kyleigh Fertitta, Pearl Thompson, Philip T. Patton, Isabelle Charrier, Stacie J. Robinson, Aude Pacini, Lars Bejder

**Affiliations:** ^1^ Marine Mammal Research Program, Hawaii Institute of Marine Biology, University of Hawaii at Manoa, Kaneohe, HI, USA; ^2^ Institut des Neurosciences, Université Paris-Saclay, Saclay, France; ^3^ Pacific Islands Fisheries Science Center, Hawaiian Monk Seal Research Program, National Oceanic and Atmospheric Administration, Honolulu, HI, USA

**Keywords:** vocalizations, combinational calls, bioacoustics, marine mammals, Hawaiian monk seal, vocal repertoire, communication

## Abstract

Hawaiian monk seals (HMS; *Neomonachus schauinslandi*) are endemic and endangered with a population of approximately 1600 individuals. While research has provided extensive information on HMS biology, movements and population ecology, its underwater vocal behaviour remains largely undocumented, with previous descriptions limited to two individuals in human care. To broaden our understanding of sound production in free-ranging seals, we deployed passive acoustic recorders at five sites across the Hawaiian archipelago. From >4500 h of recordings, we manually detected and classified >23 000 underwater vocalizations. A discriminant function analysis of 10 call types yielded an average correct classification rate of 63%. We identified 25 call types, including five published elemental calls and 20 novel calls. Nineteen of the novel call types were combinational calls—an undocumented communication strategy in pinnipeds. The novel *Whine*, captured via biologging tag- and citizen-scientist videos, provided a rare example of context-specific call use in pinnipeds. Vocalizations were low frequency (<1 kHz), short–medium duration (<7 s), with 66% occurring in bouts. Calls were detected throughout the day at three of five sites, with peaks at night and late afternoon. These findings establish a baseline for HMS vocal behaviour and emphasize the importance of acoustic communication in future research and conservation efforts.

## 1. Introduction

Acoustic communication is a fundamental aspect of animal behaviour, enabling individuals to convey critical information for survival, reproduction and social interactions [1]. In the marine environment, where light attenuation limits visual cues, sound propagates efficiently over long distances [2]. Marine mammals, including cetaceans and pinnipeds (i.e. true seals, fur seals, sea lions and walrus), rely heavily on acoustic signals to navigate, locate prey and interact socially. Pinnipeds are unique among marine mammals as they are amphibious and therefore require effective acoustic communication both on land and under water. Among phocids (i.e. true seals) that mate aquatically, males produce underwater vocalizations during the breeding season associated with reproductive displays, including for male–male competition and mate attraction [3–5].

Vocal repertoires, acoustic structure of calls, spatial-temporal patterns and behavioural functions of vocalizations are well documented for several phocid species (reviewed by [6]). For example, adult male harbour seals (*Phoca vitulina*), bearded seals (*Erignathus barbatus*), and various Antarctic seals produce vocalizations such as roars, trills and hoots during their respective breeding seasons [7–9]. By contrast, the vocal behaviour of monk seals (*Monachus* and *Neomonachus* spp.), the only phocid lineage adapted to tropical and subtropical environments, remains poorly studied [10]. Understanding the acoustic ecology of these Vulnerable or Endangered seals [11,12] is critical for conservation, particularly in regions where anthropogenic noise may mask acoustic communication.

The Hawaiian monk seal (HMS; *Neomonachus schauinslandi*) is an endangered species endemic to the Hawaiian archipelago, with an estimated population of approximately 1600 [13]. Unlike temperate and polar phocids, which have temporally restricted breeding seasons [4], HMS inhabit a stable, warm environment where females can give birth year-round [14]. This protracted reproductive cycle supports an atypical breeding strategy, in which males serially compete for access to females throughout the year [15–19].

While earlier studies documented HMS airborne vocalizations, including mother–pup contact calls and adult social calls [15,20–22], their ability to produce and receive sound underwater remained unexamined until recently. The first published underwater audiogram for HMS showed they were insensitive to low-frequency sounds, suggesting they were unable to detect their own vocalizations and most anthropogenic sounds [23]. By contrast, recent underwater audiograms for two HMS demonstrated that this species can hear over a wider range of frequencies than previously measured—with highest sensitivity between 0.1 and 40 kHz—encompassing the frequencies of their underwater vocalizations [24,25].

The first detailed descriptions of HMS underwater vocalizations revealed a repertoire of six low-frequency vocal types in two captive adult males [24,26]. These studies found a strong relationship between vocal behaviour and testosterone levels in one individual, suggesting a reproductive function for calling, even in the absence of conspecifics. They also found an extended period of heightened vocal activity, indicating a protracted breeding season [24,26]. While these studies provided foundational insights into HMS vocal behaviour, they were limited to two individuals in human care.

Until recently, reports of underwater vocalizations by free-ranging HMS were limited to anecdotal accounts of *foghorn* and *bark* calls [17]. A recent soundscape analysis confirmed the presence of HMS underwater vocalizations at four sites throughout the Hawaiian archipelago, demonstrating that free-ranging individuals produce underwater vocalizations [27]. However, these studies did not characterize vocalization types—a critical first step in understanding a species’ communication system [6]—nor did they examine temporal patterns in sound production in relation to the breeding season.

Passive acoustic monitoring (PAM) is used to study marine mammal occurrence, distribution, behaviour, population structure, abundance and ecology [28,29]. In addition to documenting biological sources of sound, PAM is increasingly used to assess the impacts of anthropogenic noise on marine life, including its potential to mask critical acoustic communication signals and alter behavioural patterns [30–35]. Because PAM allows for long-time continuous monitoring in remote or difficult-to-access habitats, it is a valuable tool for evaluating how noise pollution affects vocal species like the HMS. This is particularly relevant as their population increases in the main Hawaiian Islands (MHI), where over 1 million people reside and where increasing human activity—including coastal development, boat traffic and tourism—may introduce chronic noise disturbances. Understanding HMS vocal behaviour through PAM is essential for assessing their resilience to changing acoustic environments and informing conservation efforts aimed at mitigating potential noise-related impacts.

To broaden our understanding of sound production for free-ranging seals, we deployed passive acoustic recorders at five sites throughout the Hawaiian archipelago. This study had three primary objectives: (i) to characterize the vocal repertoire and compare it with the published repertoire of captive seals, (ii) to examine diel patterns in calling behaviour, and (iii) to compare the number of calls across geographic sites. By providing the first quantitative assessment of free-ranging HMS underwater sound production, this study establishes a foundation for future research on their acoustic ecology and informs conservation strategies for this endangered species.

## 2. Material and methods

### 2.1. Study sites

**Figure 1.**
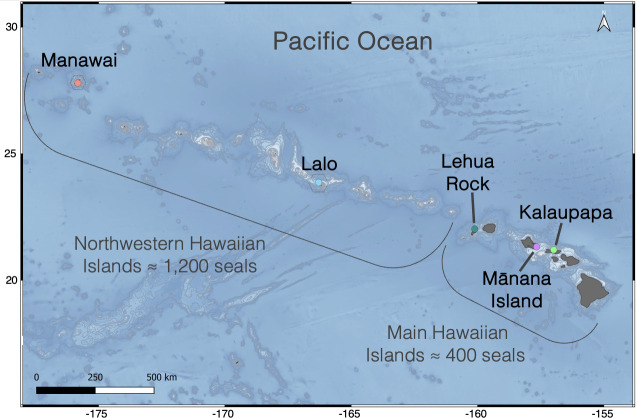
Map of the Hawaiian archipelago showing SoundTrap deployment locations within five Hawaiian monk seal critical habitats. Locations include Kalaupapa, Molokaʻi; Mānana Island, O‘ahu; and Lehua Rock in the main Hawaiian Islands, and Tern Island, Lalo, and Southeast Island, Manawai in the Northwestern Hawaiian Islands. Image created in QGIS.

Acoustic recorders were deployed at five sites in the Hawaiian archipelago between 2020 and 2023. Three sites were in the MHI: Mānana Island, Oʻahu; Lehua Rock, Niʻihau; and Kalaupapa, Molokaʻi. Two additional sites were in the Northwestern Hawaiian Islands (NWHI) within the Papahānaumokuākea National Marine Sanctuary: Tern Island, Lalo; and Southeast Island, Manawai (figure 1, table 1). These areas are recognized as critical terrestrial and aquatic habitats of the HMS [36]. Detailed descriptions of these recording sites can be found in Parnell *et al.* [27].

**Table 1.** Summary of acoustic monitoring efforts across five underwater critical habitats of the Hawaiian monk seal. The Lehua Rock recorder was deployed at a deeper depth due to the site’s bathymetry and was damaged after 7 d of deployment.

site name	GPS	depth (m)	deployment dates	no. days recorded	no. days analysed	no. hours analysed	time analysed for diel patterns (h)	time analysed for vocal repertoire (h)
Mānana1	21°19.503 N	7.9	22 July 2020–18 Aug 2020	28	28	642	54	642
	157°39.624 W							
Mānana2	21°19.611 N	6.7	22 July 2020–18 Aug 2020	28	28	642	54	642
	157°39.689 W							
Mānana3	21°19.612 N	6.7	31 Aug 2021–20 Oct 2021	51	51	1198	100	1198
	157°39.688 W							
Lehua	22°00.881 N	19.8	10 May 2021–28 May 2021	7	7	148	12	12
	160°06.194 W							
Kalaupapa	21°12.218 N	5.5	26 July 2023–12 Oct 2023	52	52	1220	102	102
	156°59.037 W							
Lalo	23°51.984 N	7.9	23 July 2021–5 Sept 2021	45	20	458	38	47
	166°17.321 W							
Manawai	27°47.523 N	8.2	8 Aug 2021–18 Sept 2021	24	11	251	21	21
	175°49.153 W							
			**totals**	**235**	**197**	**4559**	**381**	**2664**

### 2.2. Acoustic data collection

Recordings were collected intermittently between 2020 and 2023 using three SoundTrap 500HF recorders (0.02–150 kHz, ±3 dB; Ocean Instruments, Warkworth, Auckland, New Zealand). The SoundTraps recorded continuously at a sampling rate of 96 kHz for all deployments except Kalaupapa, where we used a 50% duty cycle at 48 kHz sampling rate to optimize battery life and storage because the retrieval date was uncertain. Each instrument was retrieved and serviced at the end of its deployment. The SoundTraps were secured horizontally to concrete blocks and placed on the seafloor, positioning the hydrophone approximately 0.15 m above the substrate. At Lalo and Manawai, a line with a buoy was attached to the block to facilitate retrieval. SoundTraps were deployed at depths ranging from 5.0 to 19.8 m (table 1). Due to logistical constraints, the deployments were not simultaneous; NWHI sites and Lehua Rock were accessible only during summer months.

### 2.3. Manual detection and classification of vocal types

Vocalizations were detected and classified through an audio-visual review of spectrograms using the Logger remora in Triton (version 1.93.20160524; Scripps Whale Acoustics Lab) within MATLAB R2018b. Spectrograms were viewed in 30 s windows with a frequency range of 0–4 kHz, employing a 16 384-point fast Fourier transform (FFT), Hanning window, and 90% overlap. We logged start and end times from the spectrogram for each detected vocalization, and classified calls as one of six published vocal types (i.e. *Croak*, *Groan*, *Growl*, *Moan*, *Rumble*, *Whoop*) [24]. Each call was scored on a three-point scale for subjective quality with ‘1’ indicating faint or low-amplitude calls and ‘3’ for loud or high-amplitude calls. This scale was used only as an initial screening step to identify calls suitable for quantitative measurement. *Croaks* were defined as calls <0.5 s, while *Growls* exceeded 0.5 s, following observed temporal differences in these vocal types [24]. When two or more of the six published vocal types were produced sequentially without ambient noise separating them, they were reviewed and combined into a single call type (e.g. *MoanGrowl*). These concatenated vocal types are referred to as ‘combinational’ calls, while the six published vocal types are referred to as ‘elemental’ calls throughout this study. Bouts were defined as two or more elemental or combinational calls separated by less than 3 s of silence, consistent with typical call spacing observed in previous studies [24,26]. Although individual identity cannot be confirmed from PAM data, we assumed bouts were produced by the same seal based on short inter-call intervals (<3 s) and consistent call structure and amplitude, following the approach of Sills *et al.* [24]. By contrast, overlapping calls of differing amplitudes suggested multiple calling individuals and were treated as separate bouts.

Vocalizations that could not be categorized as one of the six known vocal types were classified as ‘unknown’ and then reviewed by a trained analyst (K.P.) to assign a new call type. Only calls that consistently occurred within or near bouts of published HMS vocalizations and exhibited similar spectral–temporal characteristics were retained and classified as HMS calls. By contrast, sounds detected in isolation or with dissimilar acoustic features (e.g. higher frequency) were excluded from further analysis. Many of these unknown sounds were identified as fish based on comparison with known soniferous Hawaiian reef fish [37].

Among the retained ‘unknown’ vocalizations, one call type was perceptually distinct from the published repertoire and was named the *Whine*. This vocalization resembled underwater calls produced by free-ranging HMS while foraging, as observed in footage from biologging tags and publicly available videos [38] (electronic supplementary material, video S1). Two additional ‘unknown’ vocalizations, the *Hum* and *Grunt*, were detected, but neither occurred in isolation. The *Hum* was only observed as a transitional vocalization between two other vocal types (e.g. *MoanHumGrowl*), while the *Grunt* was only produced following *Whoops*. Because they never occurred independently, they could not be spectrographically analysed as separate call types (i.e. elemental calls) and are not included in the overall vocal repertoire count.

To facilitate efficient detection and classification of the acoustic data, we used representative sound files and spectrographic exemplars from Sills *et al.* [24] to train analysts who examined the datasets. Upon completion of a dataset, a lead analyst (A.D., C.M.S., P.T., K.P.) reviewed all logged vocalizations to confirm classification accuracy.

The amount of data analysed at each site varied based on the total recording duration and the vocalization rate (table 1). At Mānana Island, all data were analysed due to the low detection rate of HMS vocalizations. At Lehua Rock and Kalaupapa, 5 min per hour were analysed for every day of the deployment. At Lalo and Manawai, 5 min per hour were analysed on 3 days per week to account for the high detection rate of HMS vocalizations. The decision to analyse 5 min per hour was based on visual inspection of pilot datasets from each site, which showed that this subsampling captured the majority of vocal activity and general patterns without significant loss of call types or diel structure. This approach also allowed for consistent comparisons across sites while balancing feasibility of manual processing.

### 2.4. Quantitative classification of vocal types

To formally characterize the vocal repertoire of free-ranging seals and compare with the published repertoire, we performed a spectrographic analysis using a subsample of at least five representative calls per vocal type. To minimize the likelihood that these calls originated from the same individual, we selected calls from different recording files spaced across time within each deployment, rather than measuring multiple calls of the same type from a single file. Where possible, we selected up to 20 calls per call type from each site to further reduce the potential for individual overrepresentation. The calls selected for quantitative analysis were those rated as high quality (‘3’) during the initial screening and that also had signal-to-noise ratios (SNRs) greater than 10.5 dB (relative units), as determined by the SNR NIST Quick measurement in Raven Pro 1.6 [39]. Spectrograms of the intended signal were bandpass filtered from 20 to 1000 Hz and viewed from 0 to 1100 Hz, with an 8192-point discrete Fourier transform, Hann window, 90% overlap, and a 3 dB filter bandwidth of 16.9 Hz. With this configuration, frequency and time resolutions were 11.7 Hz and 0.0085 s, except at Kalaupapa (48 kHz sampling rate; 19 calls analysed), where they were 5.9 Hz and 0.017 s, respectively. Twelve spectral and temporal parameters were measured for each vocalization using Raven Pro 1.6. The parameters measured (see descriptions and abbreviations in table 2) were SNR NIST Quick, total duration, 90% duration, centre frequency, 50% bandwidth (and the 25% and 75% frequencies), 90% bandwidth (and the 5% and 95% frequencies), peak frequency and aggregate entropy.

**Table 2.** Mean (± s.d.) values of acoustic parameters measured for 10 vocal types produced underwater by free-ranging Hawaiian monk seals and included in the leave-one-out cross-validated discriminant function analysis (LOOCV DFA). Only high-quality calls with signal-to-noise ratios > 10.5 dB were analysed, and call types were included only if ≥5 measured calls were available. All parameters were measured using Raven Pro 1.6 software (Hann window; 8192-point discrete Fourier transform; 90% overlap; 3 dB filter bandwidth 16.9 Hz). Dashes (—) indicate parameters that were not measured for a particular vocal type. Asterisks (*) denote descriptive features measured only for certain vocal types. Bolded parameters were used as predictors in the LOOCV DFA.

parameter	definition	Croak	Growl	GrowlRumbleWhoop	Moan	MoanGrowl	MoanGrowlRumbleWhoop	MoanHumGrowl	MoanWhine	MoanWhoop	Whoop
		*n* = 82	*n* = 102	*n* = 7	*n* = 40	*n* = 24	*n* = 5	*n* = 5	*n* = 8	*n* = 7	*n* = 114
**total duration (s) (DUR)**	duration of the complete call	0.33 ± 0.12	2.94 ± 1.44	3.25 ± 1.35	0.67 ± 0.32	4.01 ± 1.54	3.00 ± 1.05	2.94 ± 0.61	1.17 ± 0.37	1.79 ± 0.80	0.14 ± 0.05
duration 90% (s) (DUR90)	duration of the call containing 90% of the total energy	0.18 ± 0.08	2.29 ± 1.17	2.76 ± 1.17	0.49 ± 0.22	3.17 ± 1.19	2.39 ± 1.16	2.43 ± 0.66	0.95 ± 0.32	1.46 ± 0.68	0.08 ± 0.03
**frequency 5% (Hz) (F5)**	lower frequency bound containing 5% of total energy	137 ± 46	138 ± 48	105 ± 23	36 ± 15	94 ± 55	77 ± 51	124 ± 29	45 ± 8	44 ± 6	162 ± 56
frequency 25% (Hz) (F25)	lower frequency bound containing 25% of total energy	198 ± 39	201 ± 34	162 ± 33	59 ± 12	168 ± 78	152 ± 85	176 ± 8	83 ± 59	74 ± 19	227 ± 52
centre frequency (Hz) (Fcenter)	frequency that divides the call’s energy into two equal parts	233 ± 46	237 ± 49	229 ± 26	78 ± 19	245 ± 79	237 ± 97	192 ± 13	132 ± 77	107 ± 27	273 ± 66
**50% bandwidth (Hz) (BDW50)**	difference between frequencies 75% and 25%	77 ± 81	79 ± 47	117 ± 19	44 ± 42	137 ± 92	159 ± 61	54 ± 47	135 ± 63	94 ± 75	95 ± 56
frequency 75% (Hz) (F75)	upper frequency bound containing 75% of total energy	275 ± 72	280 ± 63	279 ± 32	103 ± 46	306 ± 91	312 ± 111	230 ± 53	218 ± 62	167 ± 89	322 ± 84
frequency 95% (Hz) (F95)	upper frequency bound containing 95% of total energy	363 ± 94	387 ± 90	410 ± 73	249 ± 202	425 ± 74	450 ± 105	293 ± 102	379 ± 126	316 ± 125	408 ± 130
**90% bandwidth (Hz) (BDW90)**	difference between frequencies 95% and 5%	226 ± 118	249 ± 97	306 ± 79	212 ± 206	331 ± 85	373 ± 137	169 ± 93	334 ± 127	273 ± 130	246 ± 138
**peak frequency (Hz) (Fpeak)**	frequency of maximum power	230 ± 63	246 ± 64	158 ± 48	80 ± 24	188 ± 104	277 ± 186	188 ± 8	151 ± 96	144 ± 118	270 ± 86
**aggregate entropy (bits) (AE)**	measure of disorder in the call’s energy distribution	4.0 ± 0.7	4.2 ± 0.7	4.9 ± 0.6	3.3 ± 0.7	4.5 ± 0.6	4.8 ± 0.5	3.6 ± 0.7	4.1 ± 0.4	4.1 ± 0.7	4.4 ± 0.6
SNR NIST Quick (dB) (SNRNIST)	signal-to-noise ratio using the NIST quick method	21 ± 3	19 ± 5	24 ± 4	22 ± 4	20 ± 4	26 ± 3	20 ± 3	19 ± 2	23 ± 3	22 ± 3
presence or absence of harmonics*	present if call contained harmonics; absent if call did not contain harmonics	absent	absent	absent	present	present	present	present	present	present	absent
number of harmonics*	number of harmonics counted in the spectrogram	—	—	—	5 ± 2	4 ± 2	4 ± 1	3 ± 2	4 ± 2	4 ± 1	—
number of whoops per bout*	total number of units in a series	—	—	2 ± 2	—	—	1 ± 0	—	—	1 ± 0	4 ± 2

All calls were manually boxed by a single analyst (K.P.) to ensure consistency. Start and end points were selected in the waveform view at the clear onset and offset of the signal above background noise, with the view zoomed in to ensure precise boundary placement. Total duration was determined directly from these manual selections, while all other parameters were automatically calculated from the spectrogram view. Descriptive features such as the presence, absence and number of harmonics in certain call types were also noted. For *Whoops* produced in bouts, the above parameters were measured from one or more high-quality *Whoops* within the bout, and the number of units per bout was noted. These quantitative and qualitative measurements were subsequently used in a discriminant function analysis (DFA) to assess classification accuracy and differences between call types.

To improve DFA reliability, we excluded SNR NIST Quick, as varying recording distances and noise levels make this measurement unreliable for characterizing vocal types. This left 11 parameters for further analysis. A pairwise Pearson correlation analysis (threshold >0.7) was used to minimize redundancy and potential multicollinearity of parameters. The parameters removed included centre frequency, frequency 25%, frequency 75%, frequency 95% and duration 90%. Aggregate entropy exceeded the threshold but was retained for its significant contribution to vocal type classification, as demonstrated in Sills *et al.* [24].

The remaining six parameters were incorporated into the leave-one-out cross-validated (LOOCV) DFA. Vocal types with <5 measured calls were excluded, leaving 10 of 25 preliminary types. The LOOCV DFA generated a confusion matrix of classification scores and statistics, indicating how well the measured call variables separated into pre-assigned vocal types. A non-cross-validated DFA was also performed to visualize the separation of vocal types in acoustic space. The contributions of the six acoustic parameters to linear discriminants (LD1 and LD2) were calculated to identify which parameters most heavily influenced the separation of vocal types. Significant differences between LD1 and LD2 were evaluated using a one-way ANOVA (not assuming equal variances). All analyses were performed in R v.4.4.1 [40] using the ‘MASS’, ‘caret’, ʻcorrplot’, ʻreshape2’, ‘ggplot2’ and ‘dplyr’packages.

### 2.5. Temporal analysis of calls and comparisons across sites

Manual call counts from the detection and classification analysis were used to summarize diel patterns in sound production at each site. Only vocalizations detected during the first 5 min of every hour were included to ensure consistent representation across sites. Diel patterns of vocal activity were quantified by calculating the mean number of calls per 5-min window for each hour across the deployment, both for the total number of calls and for the four most frequent vocal types. This approach allowed for comparison of calling patterns across sites.

To quantitatively compare daytime versus nighttime patterns, all analysed hours were classified as day or night using site-specific sunrise and sunset times (HST). For hours with no detected calls, and thus not classified as day or night, we applied a fixed split (06.00–17.59 as day; 18.00–05.59 as night). Twilight was not analysed separately because coverage was limited and the period is brief, keeping effort comparable and estimates stable. We report the mean ± standard error number of calls per 5-min window. Differences between day and night were tested within each site using a Wilcoxon rank-sum test (counts are non-normal and zero-heavy); *p*-values were Benjamini–Hochberg adjusted to control the false discovery rate across sites. Analyses were conducted in R v.4.4.1 [40] using ‘ggplot2’, ‘dplyr’, ‘lubridate’, ‘tidyverse’ and ‘readr’ packages.

## 3. Results

### 3.1. Underwater vocal repertoire

**Figure 2.**
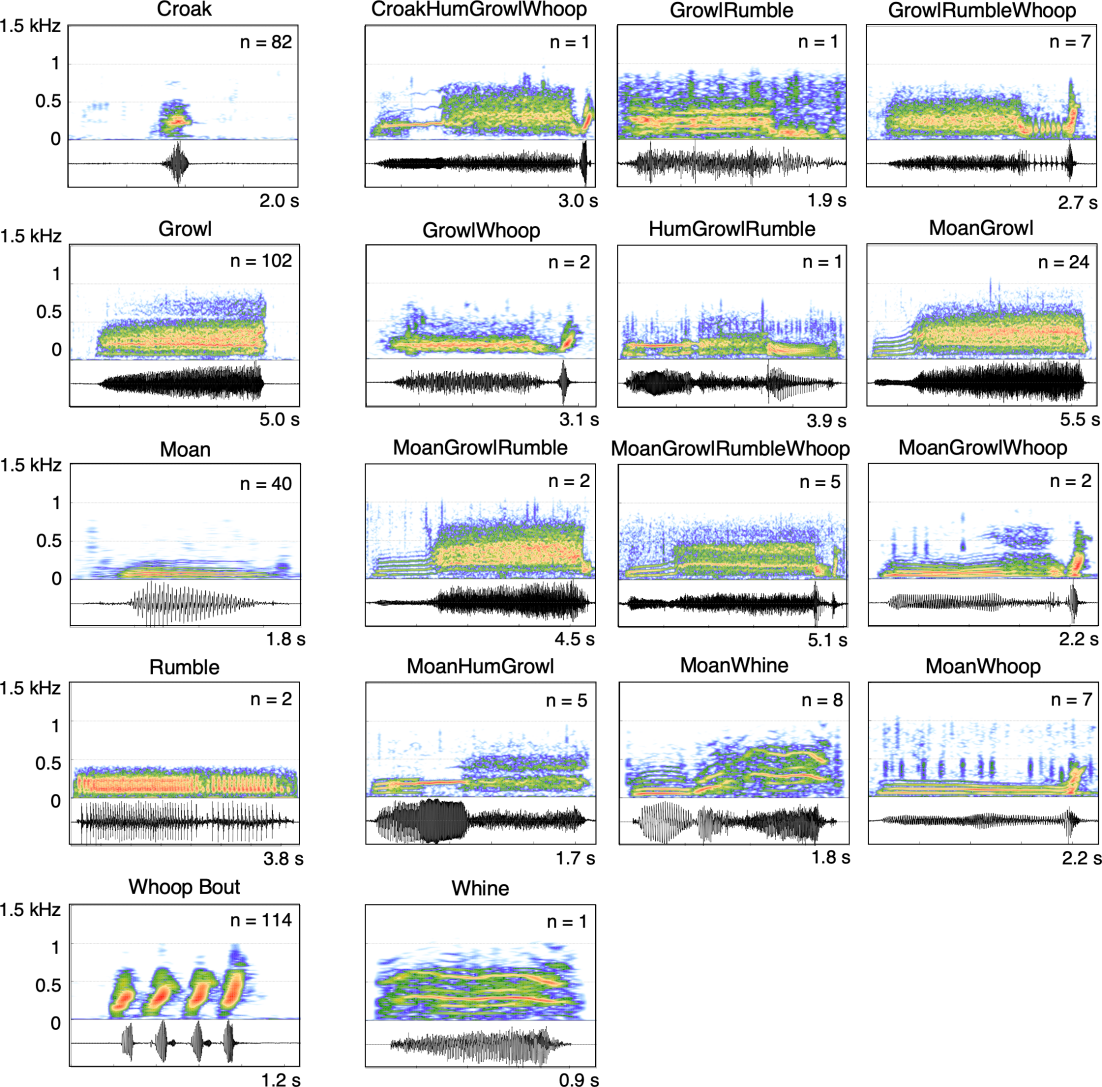
Spectrograms and waveforms of 18 underwater vocal types produced by free-ranging Hawaiian monk seals (electronic supplementary material, audio S1), each with at least one exemplar >10.5 dB signal-to-noise ratio. Elemental calls (left column: *Croak*, *Growl*, *Moan*, *Rumble* and *Whoop*) were previously described for two seals in human care [24]; all other spectrograms are novel combinational or elemental call types. The number of high-quality calls measured is displayed in the upper right corner of each spectrogram. Spectrogram settings: Hann window, 8192-point FFT, 90% overlap, except *Moan* (16 384-point FFT) and *Rumble* (4096-point FFT).

A total of 25 vocal types were identified through audio-visual categorization during the detection and classification process (table 3, figure 2; electronic supplementary material, audio S1). These included five of the six published call types [24,26], which are considered ‘elemental’ vocalizations (single vocal units separated by silence, following the criteria outlined in Sills *et al.* [24]). One novel elemental call type, the *Whine*, was identified. Additionally, 19 novel ‘combinational’ calls were identified, consisting of two or more elemental calls produced sequentially without silence separating them (e.g. *MoanGrowl*, *GrowlRumbleWhoop*). Two additional novel sounds, the *Hum* and *Grunt*, were occasionally embedded within combinational calls but were never produced in isolation and therefore were not counted in the overall vocal repertoire. Of the 25 identified vocal types, only 10 had at least five high-quality exemplars (high SNR) suitable for acoustic analysis and inclusion in the DFA.

**Table 3.** Number and percentage of total Hawaiian monk seal underwater vocalizations detected for 25 vocal types across five sites. M1–M3 indicate deployments at Mānana Island. Bolded vocal types were included in the leave-one-out cross-validated discriminant function analysis. Percent of total calls is shown in the rightmost column; rare call types (<0.1%) are marked ‘—’. *Hum* and *Grunt* were not analysed individually as they never occurred as discrete calls.

vocal type	M1	M2	M3	Lehua	Kala	Lalo	Manawai	total	% of total calls
**Croak**	5	24	36	1904	6	4535	1702	**8212**	35.1%
CroakHumGrowlWhoop	0	0	0	0	0	0	1	**1**	—
**Growl**	17	60	68	2847	4	5249	2318	**10 563**	45.1%
GrowlRumble	0	0	1	0	0	0	2	**3**	—
**GrowlRumbleWhoop**	0	1	4	0	1	3	4	**13**	0.1%
GrowlWhoop	0	4	1	4	0	3	3	**15**	0.1%
HumGrowl	0	0	0	0	0	0	1	**1**	—
HumGrowlRumble	0	0	0	0	0	0	1	**1**	—
HumWhoop	0	1	0	0	0	0	0	**1**	—
**Moan**	0	1	4	69	2	204	137	**417**	1.8%
**MoanGrowl**	0	2	4	30	1	132	106	**275**	1.2%
MoanGrowlMoanWhoop	0	1	0	0	0	0	0	**1**	—
MoanGrowlRumble	0	0	0	0	0	0	2	**2**	—
**MoanGrowlRumbleWhoop**	0	2	1	0	0	0	2	**5**	—
MoanGrowlWhoop	0	0	2	0	0	1	1	**4**	—
MoanHum	0	0	0	0	0	0	2	**2**	—
**MoanHumGrowl**	0	0	0	0	0	0	26	**26**	0.1%
MoanHumGrowlRumbleWhoop	0	0	0	0	0	0	1	**1**	—
**MoanWhine**	0	0	0	8	0	0	0	**8**	—
**MoanWhoop**	0	1	7	12	0	7	4	**31**	0.1%
Rumble	0	0	1	0	1	0	0	**2**	—
RumbleWhoop	0	0	1	0	0	0	0	**1**	—
Whine	0	0	0	1	0	0	0	**1**	—
**Whoop**	6	110	93	647	5	2043	896	**3800**	16.2%
WhoopGrunt	0	0	0	0	0	30	0	**30**	0.1%
**total**	**28**	**207**	**223**	**5522**	**20**	**12 207**	**5209**	**23 416**	

**Figure 3.**
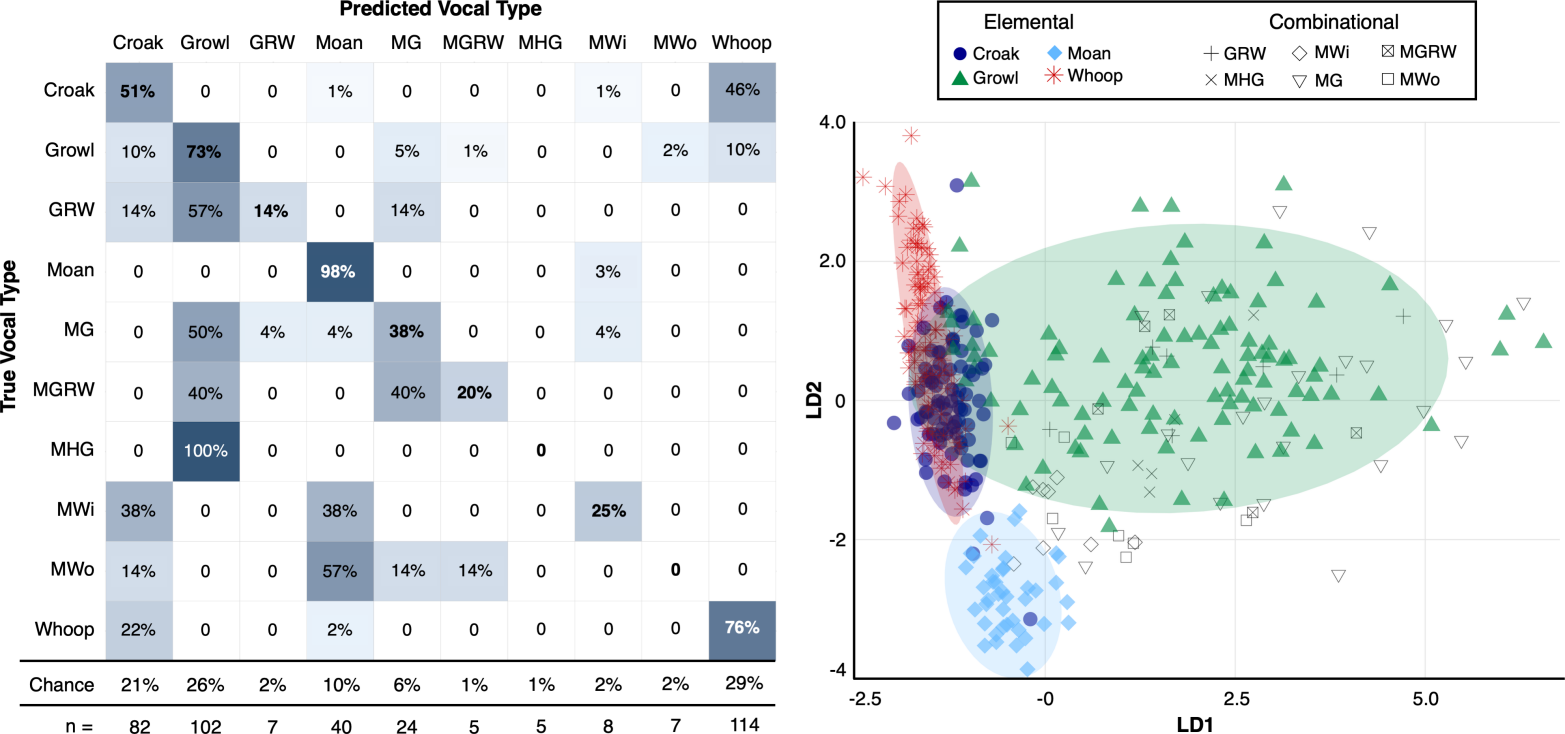
Left panel: confusion matrix for the leave-one-out cross-validated discriminant function analysis of 10 underwater vocal types produced by Hawaiian monk seals. Darker shading indicates higher classification rates; exact values are shown within each cell, with bolded numbers denoting correct classification percentages. All vocal types were classified above chance, except *MoanHumGrowl* (MHG) and *MoanWhoop* (MW). Right panel: linear discriminant plot showing separation of calls in acoustic space based on six measured parameters (see table 2). Ellipses represent 95% confidence regions for the elemental vocal types. Abbreviations: GRW = *GrowlRumbleWhoop*, MG = *MoanGrowl*, MGRW = *MoanGrowlRumbleWhoop*, MHG = *MoanHumGrowl*, MWi = *MoanWhine*, MWo = *MoanWhoop*.

The LOOCV DFA revealed moderate separation among the 10 vocal types (*Croak*, *Growl*, *GrowlRumbleWhoop*, *Moan*, *MoanGrowl*, *MoanGrowlRumbleWhoop*, *MoanHumGrowl*, *MoanWhine*, *MoanWhoop* and *Whoop*) based on six measured acoustic parameters. The DFA achieved 63% classification accuracy (95% CI: 59.8–69.4%) with a Kappa value of 0.52, indicating moderate agreement between predicted and actual vocal types (figure 3). Correct classification rates were higher for elemental call types (average accuracy: 74.5%), particularly for *Moans* (98% correct classification, chance level 10%, *n* = 40 calls), *Growls* (73% correct classification, chance level 26%, *n* = 102 calls) and *Whoops* (76% correct classification, chance level 29%, *n* = 114 calls). However, *Croaks* (51% correct classification, chance level 21%, *n* = 82 calls) were frequently misclassified as *Whoops* (46%). The six combinational calls included in the DFA were frequently misclassified as one of the elemental call types within the vocalization. For example, the *MoanGrowl* (38% correct classification, chance level 6%, *n* = 24 calls) was misclassified as *Growl* in 50% of cases. All call types were classified at rates exceeding chance levels, except for *MoanHumGrowls* and *MoanWhoops*, which had chance levels of 1% and 2%, respectively.

In the non-cross validated DFA, the first two linear discriminants (LD1 and LD2) captured 94.4% of the variance among the vocal types (LD1: 65.7%, LD2: 28.7%) (figure 3). LD1 primarily separated vocal types based on temporal characteristics, with total duration contributing 93%. LD2 was dominated by aggregate entropy (89%). Together, LD1 and LD2 provided strong separation among elemental vocal types apart from the overlap of *Croaks* and *Whoops*. By contrast, combinational calls showed substantial overlap with elemental calls. ANOVA results confirmed that both LD1 and LD2 significantly differentiated the 10 vocal types (*F*-value = 114.4 and 49.96, respectively; *p* < 0.001). These results support the clustering patterns observed in the DFA plot, where *Moan* and *Growl* formed distinct, well-separated clusters. By contrast, *Croak* and *Whoop* showed greater overlap, likely due to their shared temporal characteristics (e.g. short duration) and similar aggregate entropy. Combinational calls containing *Moans* or *Growls* tended to cluster near these two elemental call groups, reflecting their acoustic similarity. These findings suggest that the acoustic parameters shown in table 2 (electronic supplementary material, table S1) effectively capture the acoustic features that differentiate HMS vocal types which are described below.

To assess whether these DFA parameters were stable across recording sites, we conducted a *post hoc* comparison of parameter variability (reported as coefficients of variation, CVs). Peak frequency and aggregate entropy were stable (median CVs ≈ 0.09), while duration and bandwidth measures were more variable (median CVs ≈ 0.23–0.26). These results indicate that the acoustic parameters included in the DFA—and reported in table 2 and electronic supplementary material, table S1—reflect call structure, with only limited contribution from site-specific recording effects.

*Croaks* (figure 2, table 2) are brief (DUR: 0.33 ± 0.12 s), low-frequency vocalizations that lack harmonics. They are relatively narrowband (BDW90: 226 ± 118 Hz) with a peak frequency of 230 ± 63 Hz. While structurally similar to *Growls*, *Croaks* are shorter in duration and are typically produced as discrete calls—apart from a single observed combinational call, *CroakHumGrowlWhoop*. Although similar in duration to *Whoops*, *Croaks* are slightly longer and exhibit both lower aggregate entropy and lower peak frequency. *Croaks* were the second most frequently detected call type, comprising 35% of all vocalizations, and were commonly produced within vocal bouts (79%).

*Growls* (figure 2, table 2) are guttural, low-frequency calls with a relatively narrow band structure (*F*_peak_: 246 ± 64 Hz, BDW90: 249 ± 97 Hz). They are the longest duration elemental call (DUR: 2.94 ± 1.44 s), lack harmonic structure and exhibit moderate aggregate entropy (AE: 4.2 ± 0.7). *Growls* were the most frequently detected call type, comprising 45% of all vocalizations. They were commonly produced as discrete calls and appeared in 13 combinational calls. *Growls* were frequently observed within vocal bouts (52%).

*Moans* (figure 2, table 2) are relatively brief (DUR: 0.67 ± 0.32 s) tonal signals, and are the lowest-frequency elemental call (*F*_peak_: 80 ± 24 Hz). They exhibit a narrow bandwidth (BDW90: 212 ± 206 Hz) with a relatively flat frequency profile and contain an average of five harmonics. *Moans* have the lowest aggregate entropy of all measured calls (AE: 3.3 ± 0.7). Discrete *Moans* comprised 1.8% of all detected calls and were frequently produced in bouts (89%). *Moans* always occurred as the leading elemental call within 10 combinational calls. One variation, the *AscendingMoan*, was previously classified as a subtype of the *Moan* in Sills *et al.* [24], and was observed in our dataset both as a discrete call type and within combinational calls. Although this variant exhibited slight upward frequency modulation (see *MoanGrowl* in figure 2), we classified all such instances as *Moans* in this study due to their similar spectral–temporal features and to maintain consistency in call categorization.

*Whoops* (figure 2, table 2) are short-duration calls (DUR: 0.14 ± 0.05 s) characterized by a steep frequency upsweep. They occur as single elements or in a series containing 2–8 repeating elements (average: 4 ± 2 *Whoops* per bout). *Whoops* were produced in a series 64% of the time, with successive *Whoops* separated by <0.5 s. Among all elemental call types, *Whoops* exhibited the highest peak frequency (*F*_peak_: 270 ± 86 Hz). They comprised 16.2% of total vocalizations and were commonly detected within vocal bouts (73%). *Whoops* consistently appeared as the terminal elemental call in 10 combinational calls and were identified within the novel *WhoopGrunt* call type. Additionally, *Whoops* occasionally overlapped with other *Whoop* bouts that differed in amplitude—potentially indicating vocal interactions between two or more seals. The longest recorded series consisted of 50 *Whoops* of varying amplitudes.

Although *Rumbles* were not included in the DFA, two examples of this previously described elemental call type were detected at Mānana Island and Kalaupapa. *Rumbles* (figure 2; electronic supplementary material, table S1) are relatively long-duration, pulsed calls (DUR: 2.21 ± 1.48 s) that lack harmonics and exhibit a low peak frequency (*F*_peak_: 108 ± 21 Hz). They have the broadest bandwidth (BDW90: 460 ± 70 Hz) and the highest aggregate entropy among all identified vocal types (AE: 5.1 ± 1.0). *Rumbles* were the least frequently detected elemental vocal type (<0.1%) and were included in seven combinational calls.

In addition to the five elemental vocal types, one novel elemental call type was identified. The *Whine* perceptually resembles the previously described *Groan* [24,26], but it reaches higher frequencies and contains fewer harmonics (electronic supplementary material, table S1). Although only one discrete *Whine* was detected and thus could not be included in the DFA, it was primarily observed as a combinational call, the *MoanWhine* (*n* = 8), and was exclusively recorded at Lehua Rock. *MoanWhines* are longer in duration (DUR: 1.17 ± 0.37 s), have a broader bandwidth (BDW90: 334 ± 127 Hz) than *Moans*, and contain an average of four harmonics.

Lastly, we identified 19 distinct combinational calls which accounted for 1.8% (421/23 416) of the total vocalizations in our dataset. Combinational calls consisted of two to five elemental call types produced in rapid succession with no silent interval between them. The most frequently detected elemental calls within combinational calls were *Moans* and *Growls*, with the *MoanGrowl* representing 65% (275/421) of all combinational calls and 1.2% (275/23 416) of all detected vocalizations. Combinational calls were recorded across all sites; however, they occurred most frequently at Lalo and Manawai (*n* = 176 and 156, respectively), collectively representing 79% of all combinational calls.

Two additional vocalizations—the *Hum* and *Grunt*—were identified exclusively within combinational calls and were not counted as independent vocal types. *Hums*—a tonal signal—were present in seven combinational calls, frequently occurring in close association with *Growls* and *Moans*. Specifically, *Hums* preceded *Growls* in five call types and followed *Moans* in three call types. *Hums* were primarily detected at Manawai, with a single occurrence of a *HumWhoop* recorded at Mānana Island (table 3). The *Grunt* was exclusively produced in combinational calls following *Whoops*. It was only observed in a single vocal bout at Lalo (table 3), suggesting that the *WhoopGrunt* may have been produced by just one individual.

**Figure 4.**
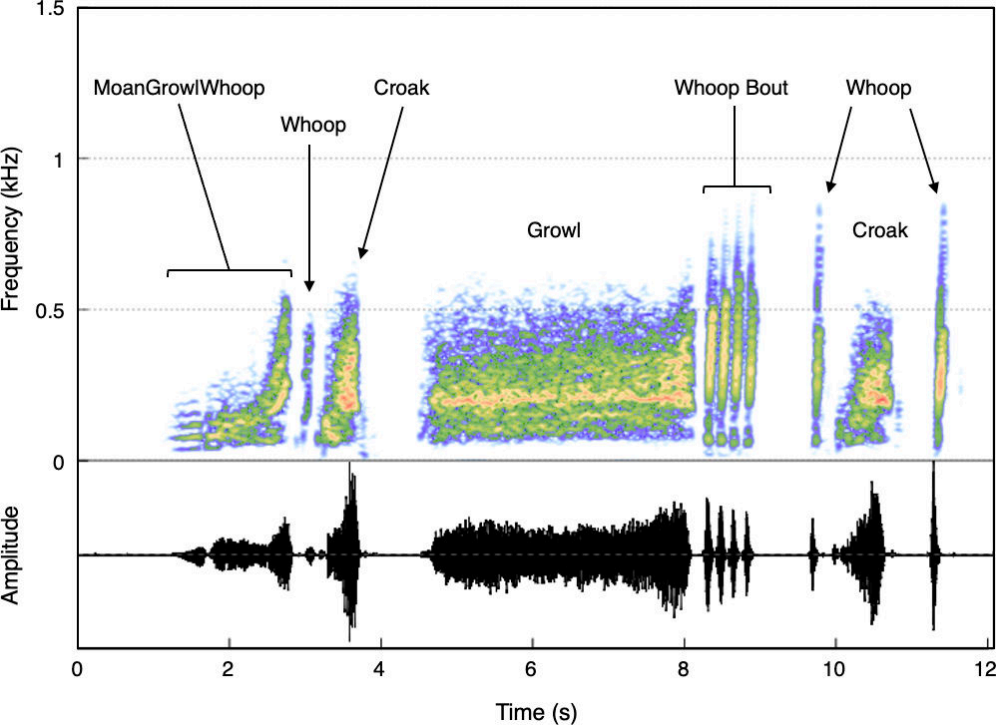
Spectrogram and waveform of an underwater vocal bout produced by a free-ranging Hawaiian monk seal at Lehua Rock (electronic supplementary material, audio S2). Spectrogram settings: Hann window, 8192-point FFT, 90% overlap.

These results confirm that free-ranging HMS produce an underwater vocal repertoire of at least six elemental vocal types, with the ability to combine them into at least 19 combinational vocalizations. Overall, 66% (15 483/23 416) of vocalizations occurred in bouts consisting of two or more elemental or combinational calls separated by less than 3 s of silence (figure 4; electronic supplementary material, audio S2).

### 3.2. Temporal patterns in vocalizations and comparisons across sites

Overall, 23 416 underwater vocalizations were detected across 4559 h of recordings (table 3). To examine diel patterns in sound production, we analysed 381 h of acoustic data (first 5 min per hour), during which 18 912 vocalizations spanning 21 vocal types were identified (electronic supplementary material, table S2).

**Figure 5.**
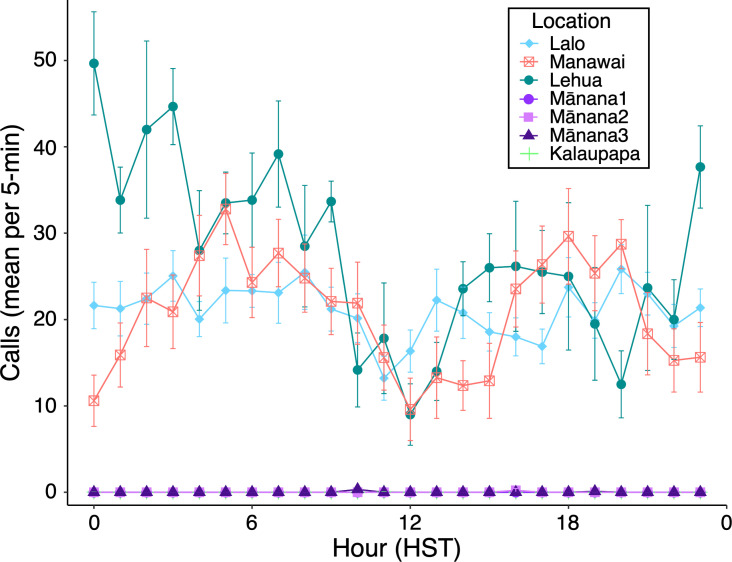
Mean (± SE) number of Hawaiian monk seal underwater vocalizations detected during the first 5 min of each hour across seven deployments at five critical habitats. Mānana1–3 and Kalaupapa had low call detections and overlap visually in the plot.

Vocalizations were detected throughout the day at high-detection sites (e.g. Lalo, Manawai, Lehua Rock), whereas sites with lower detections (e.g. Mānana Island, Kalaupapa) exhibited few or no vocalizations within the analysed 5-min intervals (figure 5). At Lalo, Manawai and Lehua Rock, the lowest number of detections consistently occurred midday (10.00–14.00 HST). Peak detections were observed between 00.00 and 05.00 HST, with a secondary peak occurring in the late afternoon (16.00–19.00 HST) (figure 5). This pattern was most stable at Lalo, where nighttime calling remained consistently high. Manawai exhibited a relative decline between 21.00 and 02.00 HST compared with Lalo and Lehua Rock. At Lehua Rock, diel variation was less pronounced: vocal activity peaked at 00.00 HST, gradually decreased throughout the day, and increased again in the early evening (14.00–18.00 HST). Overall, call detections at Lalo, Manawai and Lehua Rock were relatively high, averaging between 21 and 27 calls per 5-min window. The highest recorded vocal activity occurred at Lalo, where 77 calls were detected within the 5-min window beginning at 18.00 HST on 26 July.

Within-site Wilcoxon tests comparing the mean number of calls per 5-min window showed higher nighttime calling at Lalo (Day 19.9 ± 0.81 versus Night 22.5 ± 0.81; BH-adjusted *p* = 0.006) and Lehua Rock (Day 24.3 ± 1.58 versus Night 31.5 ± 2.35; BH-adjusted *p* = 0.049). Differences at Manawai and Kalaupapa were not significant (adjusted *p* = 0.998 for both). Mānana1 had no detected calls in the analysed 5-min windows (i.e. test was not applicable), and Mānana2 and Mānana3 had very low means (<0.03 calls per 5-min window) with no detectable day–night difference (adjusted *p* = 0.629 and 0.998, respectively).

**Figure 6.**
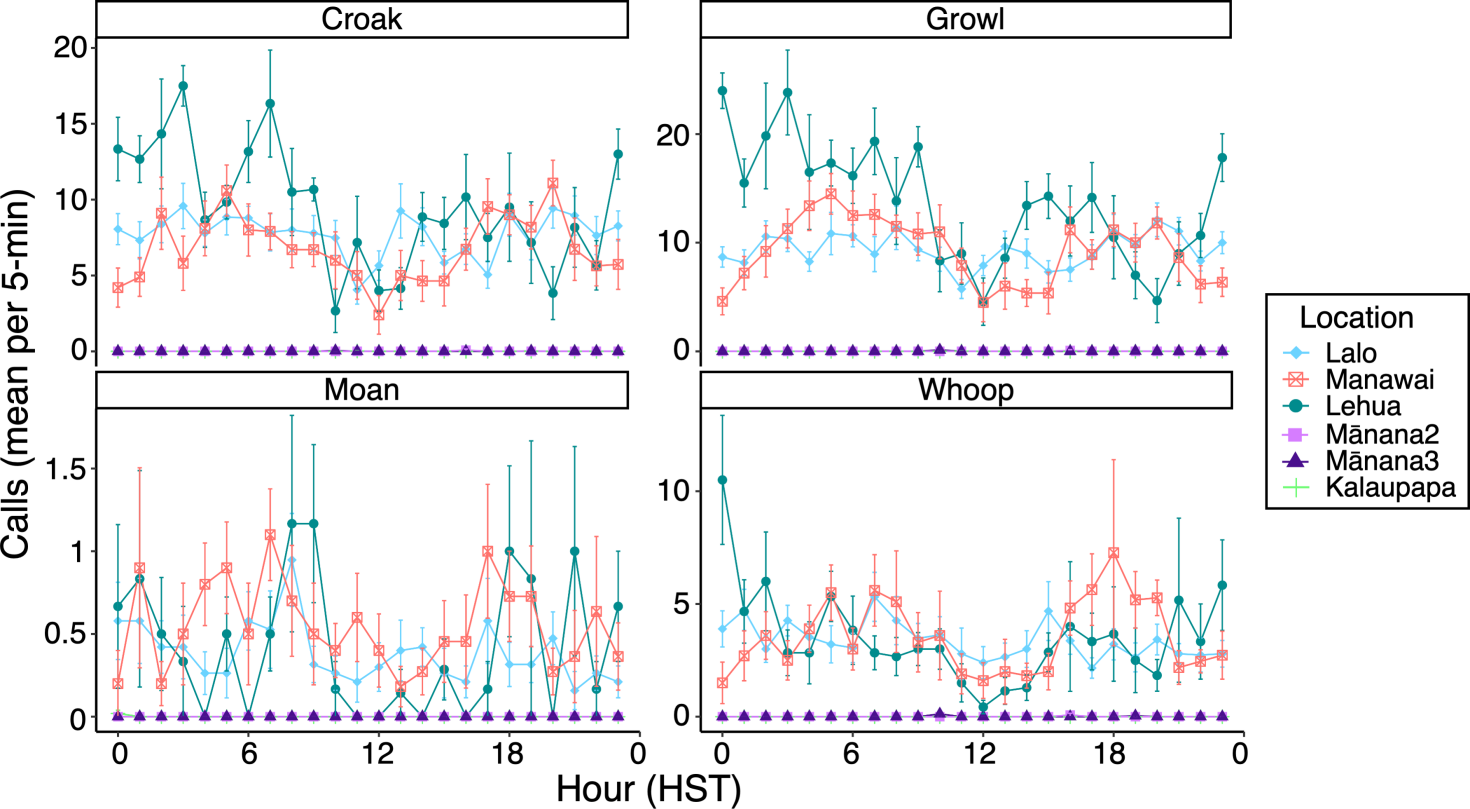
Mean (± SE) number of the four most frequent Hawaiian monk seal vocal types detected during the first 5 min of each hour. Mānana1 had no detected calls in the analysed windows and is not shown. Mānana2–3 and Kalaupapa had few detected calls and overlap visually in the plot. Note the *Y*-axis scale among vocal types, reflecting variation in overall call detections.

Patterns of diel variation differed among the four most commonly detected vocal types (figure 6). *Croaks* and *Growls* were produced consistently throughout the night, peaking between 01.00 and 05.00 HST, and gradually declined after sunrise. *Moans* and *Whoops* exhibited more variable patterns, with *Moans* peaking between 07.00 and 09.00 HST, whereas *Whoops* were more evenly distributed across day and night, with slightly higher activity in the early morning and late afternoon. These trends were consistent across high-detection sites but were difficult to assess at low-detection sites due to the limited number of recorded calls.

## 4. Discussion

This study builds upon the foundation of captive HMS research [24,26] by examining underwater sound production in free-ranging HMS and describing a diverse repertoire of calls produced throughout the day. Our findings establish that underwater vocalizations are a fundamental component of HMS at-sea behaviour, reinforcing the importance of considering acoustic signalling in conservation and management efforts for this endangered species.

### 4.1. Free-ranging and captive Hawaiian monk seals share a similar vocal repertoire

We identified 25 distinct vocal types, including five of the six call types previously described from seals in human care [24,26]. These five vocal types closely resembled those described in prior studies, with similar acoustic properties such as peak frequency and duration. The *Groan* was the only published underwater call type not detected in this study, likely because it is primarily used as an airborne signal produced by adult males and females while hauled out on beaches [15,20,41]. The most frequently produced vocal types were *Croaks*, *Growls*, *Moans* and *Whoops*, collectively accounting for 97.6% of all detected vocalizations. These call types also dominated the published repertoire [24,26].

In addition to the previously documented elemental vocal types, one novel elemental vocalization—the *Whine*—was identified. The *Whine* was not reported in previous studies of HMS in human care [24,26], potentially due to differences in environmental conditions and behavioural opportunities between captive and wild settings. Preliminary observations from paired video and acoustic data of free-ranging seals suggest the *Whine* is produced during foraging activity and associated prey capture attempts [38] (electronic supplementary material, video S1). Further evidence supporting this behavioural association is discussed in §4.4. The *Hum* and *Grunt* were also absent in the published repertoire [24,26]. These call types were rare in the dataset, perhaps reflecting limited sampling effort, specialized behavioural contexts or seasonal patterns, highlighting the need for further investigation into their behavioural and ecological significance.

Lastly, and important to highlight, we identified 19 combinational calls which accounted for only 1.8% of the total vocalizations in our dataset. Although combinational calls have been mentioned in previous studies of captive seals, they were not explicitly analysed or quantitatively characterized. Parnell [26] and Sills *et al.* [24] noted that *Moans* and their variation, the *AscendingMoan*, commonly precede and are attached to *Growls* and *Rumbles*. A more recent evaluation of the full captive dataset (>16 000 h)—of which Parnell [26] and Sills *et al.* [24] processed only a subset—similarly revealed that elemental calls are often produced continuously without silence between call types (J. Sills, personal communication). These findings suggest that HMS produce multi-component vocalizations by flexibly combining six elemental call types (i.e. *Croak*, *Growl*, *Moan*, *Rumble*, *Whoop*, *Whine*) in various uninterrupted sequences. To our knowledge, this ability to combine discrete call types into a single continuous vocalization is undocumented in pinnipeds. This communication strategy may allow HMS to convey a broader array of messages, effectively expanding their vocal repertoire.

Overall, the vocal repertoire of free-ranging HMS closely resembles that of individuals studied in human care, where opportunities for socialization with conspecifics and foraging were absent. The key exception is the *Whine*, which was absent in captive studies [24,26] but recorded in the wild, where seals engage in natural foraging behaviours. This finding highlights the influence of ecological context on vocal behaviour and suggests that certain call types may be produced only in response to specific environmental or behavioural conditions.

### 4.2. Hawaiian monk seals vocalize throughout the day

Hawaiian monk seals vocalize throughout the day, exhibiting only subtle variations in calling activity. Across the three high-detection sites (Lalo, Manawai, Lehua Rock), the fewest calls occurred midday (10.00–14.00 HST), with increases observed at night (00.00–05.00 HST) and in the late afternoon (16.00–19.00 HST). Although day–night differences were significant at Lalo and Lehua Rock, no strong or consistent diel pattern was evident across all sites. This contrasts with other phocid species, which display pronounced trends in vocal activity often related to haul-out behaviour and reproductive displays during the breeding season. For example, leopard seals (*Hydrurga leptonyx*), harp seals (*Pagophilus groenlandicus*) and crabeater seals (*Lobodon carcinophagus*) call more frequently at night, likely due to their peak haul-out period occurring during the day [42–44]. Bearded seals also produce more vocalizations at night, with peak vocal activity coinciding with the time when most females are in the water [45]. Although diel haul-out behaviour has not been explicitly quantified in relation to vocal activity for HMS, previous studies suggest they exhibit flexible and variable haul-out patterns [46]. The absence of a consistent diel trend in calling activity indicates that HMS remain active and social throughout the day and night, highlighting the importance of vocal communication in their daily behaviour.

The lack of a strong diel pattern across sites may in part reflect differences in recording effort and timing, as shorter deployments (e.g. 6 days at Lehua Rock) and low call detections at some sites (e.g. Mānana Island and Kalaupapa, both recorded outside of peak breeding season) provided limited data on diel calling behaviour. Observed diel variation may also have been influenced by changes in ambient noise levels or shifts in the position of vocalizing animals relative to the recorders, which could affect detection probability independent of calling behaviour. Previous soundscape analyses at these sites showed that ambient noise in the 250 Hz octave band was elevated during the day at Mānana Island and Lalo due to unidentified fish species, and that ecotourism vessels and biological sounds contributed to increased daytime levels at Lehua Rock [27]. These local soundscape conditions may therefore contribute to the observed diel variations in detections.

### 4.3. Detections vary by recording site

The number of calls detected varied across recording sites, with the highest vocal activity observed at Lalo, Manawai and Lehua Rock, and the lowest at Mānana Island and Kalaupapa. These patterns appear to reflect local HMS population estimates. For example, in 2019, Lalo, Manawai and Niʻihau/Lehua Rock had estimated populations of 221, 141 and 154, respectively [11]. By contrast, the MHI—including Mānana Island and Kalaupapa—had only 195 seals in 2020, distributed across the six main islands [47]. High-density sites averaged 21–27 calls per 5-min window, suggesting that the number of vocalizing individuals likely influences local detection probability. Increased social interaction opportunities at high-density sites may drive elevated vocal activity, while individual variation in vocal behaviour may also contribute to observed differences across locations.

While population size likely influences detection probability, other factors such as the recorder placement and recording period may also contribute to site differences. With the exception of Lehua Rock, recorders were deployed in shallow nearshore waters (<8.3 m), where male HMS are frequently observed ‘patrolling’ beaches while emitting in-air vocalizations [19,21]. This suggests males utilize shallow-water habitats for airborne acoustic communication, while underwater displays may occur elsewhere. For example, at Kalaupapa, scuba divers have observed large males vocalizing in deeper waters during peak pupping season (G. Puig-Santana, personal communication). Additionally, the timing of recording relative to the HMS breeding season may have influenced calling activity. Previous studies have shown that male HMS vocalizations increase with breeding activity, likely in association with reproductive behaviours [24,26]. However, recordings at Mānana Island and Kalaupapa occurred between July and October, after peak breeding months [14,20]. This timing may partially explain the decreased detections observed at these two sites.

### 4.4. Vocalizations are used for reproductive and foraging purposes

The underwater vocal behaviour of HMS is likely related to reproductive functions, similar to other aquatically mating phocids. In many species, males produce underwater vocalizations during the breeding season to attract mates, establish dominance over rivals or defend territories [4,17,48]. HMS are known to mate at sea [19], further supporting the role of underwater vocalizations in reproductive behaviours. Studies with one adult male HMS in human care revealed that vocal activity is closely associated with testosterone levels, further suggesting a reproductive function for these calls [24,26]. Sustained vocal activity observed throughout the day during the breeding season at three sites indicates that acoustic communication plays an important role in HMS social interactions. If vocalizations function for reproductive purposes, this could explain the increased number of detections documented at sites with larger HMS populations, where male–female interactions are likely frequent. Understanding the role of underwater calling is a necessary step towards evaluating the potential impacts of anthropogenic noise on acoustic communication in this endangered species, even if existing recordings may already reflect some level of exposure.

One intriguing finding of this study was the detection of the *Whine*, a vocalization not previously documented in human care [24,26]. Recent analyses of paired video–audio data from biologging tags and publicly available videos shows that both male and female seals produce this vocalization in apparent foraging contexts—typically while inverted with their heads near coral or substrate and engaging in prey capture attempts [38] (electronic supplementary material, video S1). While more detailed analysis is forthcoming, these observations suggest a potential novel function of vocal behaviour in pinnipeds—underwater calling for foraging purposes. Although rare, similar behaviour has been observed in sub-adult male northern elephant seals, which produce infrasonic sounds while pursuing prey [49]. Together, these observations raise the possibility that two species from the Monachinae subfamily produce underwater vocalizations in foraging contexts. Further investigation examining the behavioural context of the *Whine* and other vocalizations produced by HMS is currently underway [38].

### 4.5. Limitations and future directions

While this study provides new insights into HMS vocal behaviour, several limitations should be considered. First, seasonal variation in vocal activity was not assessed, preventing a direct comparison between vocal activity and breeding periods. Given that previous studies in human-care settings have linked HMS vocalizations to reproductive activity [24,26], long-term acoustic monitoring is needed to determine whether seasonal patterns in vocal activity align with reproductive cycles in the wild. Captive data suggest that vocalizations occur year-round [24,26], so we expect calling to persist across seasons for wild seals, although the rate and function of these calls may vary. Additionally, recordings were limited to a single year in the NWHI and at Kalaupapa, preventing assessment of interannual variability. Expanding PAM efforts across years and locations would provide a more comprehensive understanding of long-term trends in vocal behaviour and potential environmental and anthropogenic influences.

The functional significance of vocalizations must also be considered in the context of their auditory sensitivity. The species’ underwater hearing range suggests they are sensitive to low-frequency anthropogenic noise [24,25]. Recent findings from Parnell *et al.* [27] indicate that vessel noise can mask HMS vocalizations, potentially reducing their effective communication range. Estimating their communication space under varying ambient noise conditions is a critical next step, particularly for understanding the potential impacts of noise pollution on reproductive and foraging behaviours.

This study demonstrates that PAM is an effective tool for studying HMS sound production. However, the vast amount of PAM data collected for this study makes manual analysis impractical. Developing automated call detectors and classifiers, trained on manually detected calls from this dataset, would greatly improve monitoring efficiency. The DFA presented here highlights that the elemental call types are acoustically distinct and classifiable based on the spectral and temporal characteristics, supporting the potential for future automated classification efforts. Additionally, as PAM lacks visual context and cannot confirm the identity of the calling individual(s), it is possible that some seals were overrepresented in the dataset, particularly at sites where individuals may repeatedly use the same area. Future work that integrates multi-sensor biologging tags and publicly available videos will offer insights into the behavioural context of calls and the identity of the caller, enabling assessment of individual variation on vocal repertoire structure, calling rates and classification accuracy. These combined approaches will enhance our understanding of underwater communication in HMS ecology and help assess the species' resilience to changing acoustic environments.

## 5. Conclusions

This study examines underwater sound production in free-ranging HMS and provides the first quantitative description of their vocal repertoire, building upon previous research that focused on a small number of individuals in human care [24,26]. By analysing over 23 000 vocalizations recorded across five critical habitats, we identified 25 distinct vocal types, including five previously described elemental call types, one novel elemental call, and 19 novel combinational calls. Our findings demonstrate that HMS are highly vocal underwater, producing low-frequency calls throughout the day, with elevated vocal behaviour at certain sites. These results emphasize the importance of underwater acoustic communication for this endangered species. Given the increasing evidence of anthropogenic noise impacts on marine mammals, this study establishes a baseline understanding of HMS vocal behaviour and highlights the importance of integrating acoustic communication in future conservation and management efforts.

## Data Availability

Data and relevant code for this research work are stored in GitHub [50] and have been archived within the Zenodo repository [51]. Electronic supplementary material is available online [52].
